# Genetic Pathways to Insomnia

**DOI:** 10.3390/brainsci6040064

**Published:** 2016-12-20

**Authors:** Mackenzie J. Lind, Philip R. Gehrman

**Affiliations:** 1 Department of Psychiatry, Virginia Institute for Psychiatric and Behavioral Genetics, Virginia Commonwealth University, Richmond, VA 23298, USA; lindmj@vcu.edu; 2 Department of Psychiatry, Perelman School of Medicine, University of Pennsylvania, Philadelphia, PA 19104, USA

**Keywords:** insomnia, genetics, genome-wide association study

## Abstract

This review summarizes current research on the genetics of insomnia, as genetic contributions are thought to be important for insomnia etiology. We begin by providing an overview of genetic methods (both quantitative and measured gene), followed by a discussion of the insomnia genetics literature with regard to each of the following common methodologies: twin and family studies, candidate gene studies, and genome-wide association studies (GWAS). Next, we summarize the most recent gene identification efforts (primarily GWAS results) and propose several potential mechanisms through which identified genes may contribute to the disorder. Finally, we discuss new genetic approaches and how these may prove useful for insomnia, proposing an agenda for future insomnia genetics research.

## 1. Introduction

Insomnia is defined in the Diagnostic and Statistical Manual of Mental Disorders (DSM)-5 as difficulty falling asleep, difficulty initiating or maintaining sleep, or non-restorative sleep at least 3 times a week for a minimum of 90 days and causing clinically significant distress [1]. Its symptoms remain an important health concern, given that one in three adults experiences at least one of the main nighttime insomnia symptoms described above [2,3,4]. Prevalence of the disorder is estimated to be between 6% and 10% [4]. Current research shows that insomnia is related to a multitude of negative mental (e.g., development of psychopathology), physical (e.g., diabetes, cardiovascular disease), and occupational health outcomes [4], supporting the need for research into etiologic factors that contribute to the disorder.

Insomnia is commonly conceptualized within a diathesis-stress framework in which it is thought to result from predisposing (vulnerability or protective traits), precipitating (initiating events that trigger the insomnia), and perpetuating (behaviors and cognitions that contribute to the maintenance of insomnia) factors [5]. While there has been considerable research on precipitating and perpetuating factors, predisposing factors have received less attention. Within this category, genetics is thought to play an important role in insomnia etiology. The insomnia genetics literature, although small compared to other psychiatric disorders, is growing. This review will present an overview of genetic methods and current research, as well as discuss potential mechanisms based on genetic results and future directions. It is important to note that phenotypic definitions of insomnia remain a problem throughout genetic studies of insomnia, as few studies utilize a phenotype that reflects the actual disorder, with most focusing on symptoms or measures of sleep quality [6].

## 2. Overview of Genetic Methodology

There are a variety of methods that can be used to understand the genetic contributions to a phenotype of interest. Brief descriptions of main methods are provided in Section 2.1, Section 2.2 and Section 2.3 (and in Table 1), but the reader is directed to additional resources for a more thorough overview of twin studies [7] and statistical genetics methods [8]. Note that this section focuses on approaches used within the insomnia genetics literature, and thus does not encompass all possible genetic or genomic methods.

### 2.1. Twin and Family Studies

Twin and family studies represent a quantitative approach that allows estimation of the relative contributions of genes and environment to a trait, based on known relationships among family members. Family studies compare family members of affected individuals to those of unaffected individuals. If a phenotype has significant genetic influence, members of an affected individuals’ family will be more likely to report experiencing the same disorder/symptoms when compared to the family members of the non-affected individuals. Further, as the genetic relationship between individuals increases, family members that are more closely related are expected to be more alike on the phenotype of interest. A major limitation of the family study approach is that family members share both genes and environment: The familial aggregation that is observed could be due to either genes or shared environment and thus cannot be narrowed down to purely genetic effects.

Twin studies, however, are able to further disentangle genes and environment by comparing correlations across identical (monozygotic: MZ) and fraternal (dizygotic: DZ) twins who have been raised together [9]. This is important, since being raised together means that the twins have experienced the same family environment, minimizing the potential contributions of family environment to phenotypic differences. Since MZ twins are assumed to share 100% of their genes, and DZ twins share, on average, 50%, similarity across twin pairs can be compared. If MZ twins are more similar (i.e., their scores on a phenotype are more highly correlated with each other) than DZ twins, then one can assume that this is due to differences in genes. The classic twin study separates variance components into additive genetic effects (A), which contribute twice as much to MZ as compared to DZ twins; common environmental effects (C), experiences that twins share that make them more similar (e.g., peer groups, parental attitudes); and unique environmental effects (E), which reflect the individuals’ unique experiences and thus contribute to between-twin differences. Further, the E component also encompasses measurement error. From these results, heritability (i.e., the proportion of variance due to genetic effects) can be calculated. It is also possible to examine dominant (non-additive) genetic effects (D), instead of C, in a twin study. Note that broad-sense heritability includes total genetic effects (both additive and dominant), while narrow-sense heritability refers to the proportion of variance due to additive genetic effects only. In the sections that follow, we are using the term ”heritability“ to refer to narrow-sense heritability unless otherwise specified. Extensions of the ACE model allow for comparison of sex differences, the modeling of multiple waves of data across time to examine latent constructs and stability (longitudinal and measurement modeling), and examination of overlap in etiologic influences across multiple phenotypes. Although twin studies can quantify relative genetic and environmental contributions, they have several limitations: It is not possible to identify specific genes, and other types of genetic effects beyond those that are additive or dominant in nature, such as epigenetics or gene-gene interactions, cannot be measured.

### 2.2. Candidate Gene Studies

Candidate gene studies represent an early approach to the identification of specific genes. In this method, genes of interest are chosen *a priori* based on existing knowledge of biology and potential mechanisms (e.g., a neurotransmitter system is chosen due to its known involvement in a process thought to be involved in the disorder; animal models indicate relevance of a certain gene). The purpose of an association study, such as a candidate gene study, is to compare variation in a gene of interest across individuals with and without the disorder (or with different levels of symptoms). The most common type of genetic variation studied is the single nucleotide polymorphism (SNP), which represents a base pair change at a specific position, occurring in some individuals but not others, creating two different alleles. On average, unrelated individuals differ at 1 bp per every 1000 bps across the genome. The allele that is more often observed in the ancestral population under study is the major allele, and the less common is the minor allele. A simple association approach in a case-control design tests if the frequency of either allele (or genotype) is increased in cases versus controls. SNPs can be found in areas of the genome that code for protein sequences (i.e., coding regions), and those that do not (i.e., non-coding regions). Another important type of variation is the variable number tandem repeat (VNTR) where a short sequence of nucleotides is repeated multiple times, and individuals can differ in terms of the length of repeats they possess.

In a candidate gene study, the chosen gene(s) of interest are genotyped for appropriate polymorphisms, and association analyses (e.g., logistic or linear regressions, chi-squared tests; depending on the outcome variable) are conducted to determine if allele or genotype frequencies are significantly related to the gene of interest. Extensions of the candidate gene approach investigate gene-environment interactions (G×E), which hypothesize that different genotypes may result in differential responses to environmental exposure and thus contribute to variation in the phenotype of interest. Stressful life events are commonly studied using a G×E approach. Despite initial popularity, the candidate gene approach has fallen out of favor, given limitations such as the choice of gene, which is limited to what is thought to be biologically plausible. The majority of candidate gene studies have also failed to replicate, which could be due to multiple reasons (e.g., phenotypic heterogeneity, false positives).

### 2.3. Genome-Wide Association Studies (GWAS)

GWAS is an association approach that allows for an unbiased interrogation of the genome, resulting in the simultaneous examination of thousands of SNPs. With recent advances in imputation techniques (which use statistical methods to infer genotypes which were not actually measured), millions of SNPs can now be studied. In a GWAS, association analyses are conducted across all available SNPs, which can be obtained in multiple ways (e.g., from a chip with a specific set of SNPs, whole genome-sequencing). GWAS is generally limited to detecting common variation. While rarer variants are often measured and/or imputed, it is difficult to examine the effects of rare variants, due to them being filtered out during quality control steps, given the lack of power to detect effects in genotyped variants at low frequencies. This may also be due to poor imputation of rare variants. There are approaches specifically for examining rare variants, but this is usually done in aggregate. With the cost of sequencing decreasing, GWAS represents a way to identify novel genes that one would not have hypothesized as being related to the trait of interest. However, the method is not without limitations, which include false positives due to the large number of tests run at once, as well as population stratification (if ancestry is not properly controlled for, results may be due to differences in the distribution of allele frequencies across populations, and not true results). In interpretation of both candidate gene and GWAS results, it is also important to consider that the identified variant may not reflect the causal polymorphism—instead, it could be in linkage disequilibrium (LD) (i.e., inherited together more often than expected by chance) with the actual causal variant. LD is a function of multiple genetic and selection processes, including time [10]. As a result, alleles are inherited in LD blocks, and those alleles that aggregate in the same block are often inherited together. Thus, instead of being the causal variant itself, a SNP could be representing, or “tagging”, other variants that are within its LD block, which are the causal polymorphisms. LD structure (i.e., size/composition of LD blocks) does differ by population, since different ancestry groups diverged at different periods of time. For example, Europeans and Asians are newer populations, so there has been less time for recombination to occur, making LD blocks larger than those of Africans.

## 3. Twin and Family Studies of Insomnia

There is a substantial twin and family literature indicating that insomnia is moderately heritable, despite varying definitions used across studies. Many investigations have focused on insomnia symptoms, as opposed to strict insomnia disorder phenotypes. Several reviews outline the genetics research on insomnia (and other sleep disorders) and provide an overview of early twin and family studies, some of which date as far back as 1966 [6,11,12]. The family study literature is small, with only five studies looking at insomnia using this method [13,14,15,16,17]. Overall, these studies support the idea that insomnia “runs in families”, but have the disadvantage of being unable to disentangle genetic and shared environmental effects. Twin studies do allow for the examination of both genes and shared environment simultaneously, since twins are assumed to have identical shared environments. There is a much larger twin literature, with over 20 studies conducted to date that include insomnia phenotypes [6,11,12]. Insomnia heritability estimates range from 22% to 59% in adults, with a much wider range for studies in children (14%–71%). The reader is directed to Table 2 for study-specific heritability estimates.

Newer twin studies of insomnia have incorporated multiple waves of data and more sophisticated models, which can provide useful information about genetic contributions to insomnia. A recent study by Lind and colleagues [31] used a large longitudinal twin sample and confirmed insomnia’s moderate heritability in adults (22%–25%) at single time points. This study was novel in that heritability estimates increased substantially when two time points were incorporated simultaneously and measurement error was removed (59% for females, 38% for males) and showed that insomnia heritability is stable across time in adults. Further, significant quantitative sex differences were demonstrated in the final longitudinal model, providing some of the first evidence for larger genetic contributions to insomnia in women (vs. men). Another recent longitudinal study twin study of insomnia examined its heritability across childhood and adolescence, utilizing four waves of data [39]. For three of the waves, the best-fit models included dominant (non-additive) genetic effects, accounting for 24%–38% of the variance across waves. Heritability was estimated at 14% at Wave 3, where the best-fit model included additive instead of dominant genetic effects. The non-additive genetic contributions to insomnia at Wave 1 contributed to insomnia across all other waves, providing evidence that genetic influences on insomnia are stable across time in youth. Additionally, there were new non-additive genetic effects at Wave 2 (approximate age: 10 years old), representing genetic innovation (i.e., new genes come into play that were not present at previous time points), but also stability, since Wave 2’s effects were also important at Waves 3 and 4. Thus, despite the inconsistency of insomnia phenotypes, findings for insomnia heritability appear to be robust, demonstrating that genetic contributions are important in the development of the disorder. However, a significant limitation of twin studies is the fact that they are unable to identify specific genes involved in a disorder; genetic contributions can only be examined in aggregate.

Given that insomnia has a bidirectional relationship with psychiatric disorders, particularly internalizing disorders [40] and is also related to many other physical health outcomes [4], there is a subset of the literature that examines the overlap between genetic and environmental influences on insomnia and other disorders and traits. Psychopathology, particularly depression and anxiety, have been examined in relation to insomnia in studies of both children and adults, and results show significant genetic overlap with insomnia [36,41,42]. There is also a small subset of the literature that examines overlap with externalizing (and other) psychiatric disorders (e.g., [42,38]). While a detailed discussion of this part of the literature will not be given here, studies demonstrating genetic overlap do have important implications for molecular genetics research, which will be covered in Section 5.

## 4. Measured Gene Studies of Insomnia

### 4.1. Candidate Gene

The candidate gene approach to identify potential genes involved in insomnia has been used throughout the past few decades. Both human and animal studies have investigated associations between insomnia/sleep characteristics and genes across various neurobiological systems. Since prior reviews have outlined the candidate gene literature for insomnia in greater detail [6,12], we will provide an overview of systems studied with a focus on the most recent literature. Initial investigations focused on genes involved in regulating circadian rhythms. Although not explicitly focused on insomnia, several of the first studies examined the role of circadian genes (*CLOCK*, *Timeless*) and sleep in individuals with mood disorders, with mixed results [43,44,45]. More recent work has been in the *Per* genes, with a Chinese study finding a significant association between *Per2* genotype and insomnia, as well as a G×E interaction with *Per2*, work stress, and insomnia [46]. Another paper, examining *Per3* and insomnia in individuals with alcohol dependence, also found a significant genotype effect [47].

One of the most common genes studied across psychiatric genetics, the serotonin transporter polymorphic region (*5-HTTLPR*), has also been analyzed with insomnia. The serotonin neurotransmitter system is particularly relevant to depression [48] in addition to having a role in sleep-wake regulation [49], making it a logical candidate gene target. Multiple studies have examined the classic *5-HTTLPR* polymorphism (short or long allele) (e.g., [50,51]), as well as polymorphisms in monoamine oxidase A (MAO-A) (e.g., [52]), which is an enzyme that breaks down serotonin, thus contributing to its availability in the brain [48]. Some of the serotonin candidate gene studies have also examined sleep in the context of psychopathology, finding significant results [53,54]. Recent studies have focused on G×E interactions, with Huang and colleagues [55] identifying significant main effects of *5-HTTLPR* (each S allele conferring a >80% increase in risk) and a significant gene-environment interaction for job-related stress, such that for individuals with the SS genotype, those with high job-related stress had higher insomnia risk and those with low job-related stress had lower risk. Further, a recent review discussing predictors of insomnia included *5-HTTLPR* as a contributor to poor sleep via stress reactivity mechanisms [56].

Other systems that have been studied in the context of insomnia, although to a lesser extent, include adenosine, GABA and orexin/hypocretin. More recent studies add dopaminergic system genes (i.e., catecholamine-*O*-methyltransferase (*COMT*); dopamine receptor D4 (*DRD4*), and dopamine transporter 1 (*DAT1*)), Apolipoprotein E (*APOE*, ε4 allele; commonly known in the literature for its role in Alzheimer’s disease), peroxisome proliferator-activated receptor-γ coactivator-1α (*PGC-1*α; important for expression of clock genes [57]), and the arylhydrocarbon receptor (*AHR*) to the list of candidate genes studied. Although there were no significant associations between the *COMT* Val158Met polymorphism and various sleep and circadian variables, including Pittsburgh Sleep Quality Index scores, in recent study of young adults [58], the researchers did find suggestive evidence for a role of the *DRD4* VNTR in daytime sleepiness that may warrant further research. An earlier study of young adults found that *DAT1*, another dopamine polymorphism, was associated with higher sleepiness [59]. Given that the dopaminergic system plays a role in sleep, this may represent a viable target system, which is highlighted in a recent review [60]. Wang & Lung [61] demonstrated that both *APOE* ε4 and *PGC-1*α polymorphisms increase insomnia risk, and that the effect of *PGC-1α* remains even after controlling for *APOE* ε4 status. Finally, *AHR* and *CLOCK* polymorphisms were studied in a sample of middle-aged women, both alone and in combination with each other. Polymorphisms across both genes decreased insomnia risk, pointing to potential protective factors [62].

Overall, the candidate gene literature for insomnia covers a wide variety of biologic systems, but few genes have been studied in detail, and findings are not often replicated. This method has several strengths, such as the biological plausibility of the gene of interest and the ability to select for functional polymorphisms, which is notably absent in more agnostic approaches. Findings must also be interpreted in the context of the method’s limitations which include bias in choice of gene (since some knowledge of biology is necessary), only genotyped polymorphisms are examined, and effect sizes of single genes are likely small and hard to detect. As new publications continue to examine novel (and existing) genes using this approach, efforts to meta-analyze across studies that examined the same candidate gene, such as the serotonin transporter, may provide a useful way to understand overall effects. However, more studies are needed across most genes in order to make this possible. A shift toward genome-wide, hypothesis-free methods is likely appropriate for insomnia genetics, as the cost of genotyping and ease of data management continue to improve.

### 4.2. Genome-Wide Association Studies

As described above, GWAS allow for the simultaneous examination of millions of variants across the genome. There are far fewer GWAS of insomnia to date than other common psychiatric phenotypes (e.g., depression [63,64], schizophrenia [65]) and sleep phenotypes such as sleep duration (e.g., [66,67]), with only four studies examining potential genes involved in insomnia or related symptomatology. Brief descriptions of insomnia GWAS (and replications) are provided in Table 3. The first GWAS of insomnia, by Ban and colleagues [68], examined insomnia based on self-report questions in a large Korean sample (*n* = 10,038). Several variants in genes previously implicated in psychiatric phenotypes (bipolar disorder, schizophrenia) were found to be of interest, rs11208305 in *ROR1* and rs718712 in *PLCB1*, although neither of these SNPs reached genome-wide significance within the sample. Next, a GWAS of multiple sleep phenotypes, including an insomnia factor score, sleep quality, and sleep latency, was conducted in a sample of Australian twins [69]. While there were once again no variants that passed the genome-wide significance threshold, the most significant result was an association between a SNP in *CACNA1C*, a gene encoding a subunit of voltage-dependent calcium channels and previously associated with bipolar disorder, with both sleep quality and latency. The association between *CACNA1C* and sleep quality was later replicated in a British cohort [70], indicating that this may be a relevant gene for insomnia. Note that within the Korean GWAS, another calcium channel gene, *CACNA1A*, was briefly discussed as a gene of interest [68]. Although neither of these studies found genome-wide significant hits, they were the first to introduce novel genes of interest via GWAS.

A recent GWAS utilized objective sleep phenotypes measured with actigraphy, in contrast to self-report measures [71]. This is a unique approach that may or may not be relevant for insomnia, given that the disorder is diagnosed based on self-report [1]. Results showed that *DMRT1* was associated with sleep latency and *ULF1* (a circadian gene) was associated with sleep efficiency on weekdays. However, note that results were not corrected for the analysis of multiple phenotypes. Finally, a new GWAS of sleep latency that meta-analyzed a combined sample of European cohorts (*n* = 4242) identified 3 correlated genome-wide significant variants within the *RBFOX3* locus [72]. These variants were able to be replicated across multiple independent populations, demonstrating the robustness of this signal. Further, functional analyses were also run and indicated a role for *RBFOX3* in GABA and monoamine signaling, and thus sleep onset. These newer GWAS have been more successful in identifying significant genes, providing additional insight into insomnia genetics.

Although GWAS results have provided us with several novel genes of interest for insomnia, the method (and interpretation of subsequent results) is not without limitations. The same genes do not emerge as significant across studies, which could be attributed, in part, to phenotypic heterogeneity, given the liberal use of the insomnia definition (to describe “insomnia” as well as sleep quality, latency, etc.) and the fact that one study used objective measures. Further, GWAS captures common variants with small effect sizes, leaving much of the heritability unexplained. While common variation is important, other types of genetic variation, such as rare variants (i.e., where the base pair change is present in less than 1% of the population), which are unable to be detected via GWAS, may also play a role. There is also the issue of multiple testing; there is a high likelihood of false positive results in GWAS given the large number of tests run at once. This is generally corrected for using a family-wise error rate, setting the significance threshold at *p* < 5 × 10^−8^, but this is a conservative approach which may result in missing important genes. Finally, power remains a problem in GWAS, as large sample sizes (for both cases and controls if the trait is binary) seem to be necessary for gene identification. The psychiatric genetics literature has made it clear that increasing sample sizes tend to produce more significant results (see schizophrenia as a good example of this; [73]), and as such, there have been many efforts to combine data through the creation of the large consortia (e.g., the Psychiatric Genomics Consortium (PGC; [74]), which began with schizophrenia, but now has many subgroups for various disorders). Combining data across research groups to create a large insomnia dataset would be useful for gene identification efforts, but the issues of inconsistent insomnia phenotyping and few actual GWAS of insomnia make this challenging in practice.

## 5. Conclusions and Future Directions

It is clear there has been progress in our understanding of the genetics of insomnia, despite limitations, and overall results do shed light on several potential molecular mechanisms for the development of the disorder. Several of the novel genes identified through GWAS also appear to be associated with other psychiatric disorders (e.g., *CACNA1C* with bipolar disorder), providing evidence for pleiotropy (where one gene can influence multiple, often unrelated, traits) and suggesting that attempts to test genes from other psychiatric disorders in insomnia may be useful. Many of the polymorphisms investigated in candidate gene studies of insomnia (e.g., serotonin transporter polymorphism, dopamine system polymorphisms) have also been examined in the context of other related psychiatric disorders such as depression and PTSD, although these disorders also report mixed findings (particularly for serotonin) (e.g., [75,76]). Further, although not identical across studies, the novel genes converge in function and suggest systems that may not have been previously considered in a candidate gene approach: Many are related to intrinsic neuronal excitability, which has a plausible role in insomnia: One study showed that individuals with insomnia had disturbed intracortical excitability [77], and some have suggested that insomnia is a result of over-activation of wake-promoting areas during sleep [78]. For example, the *CAC1AIC* gene codes for a subunit of voltage-dependent calcium channels, which are important for neuronal excitation and the release of neurotransmitters, among other functions [79]. *RBFOX3*, the main gene of interest from the sleep latency GWAS, was found to be related to the function of neurotransmitters such as GABA [72]. GABA is the main inhibitory neurotransmitter in the brain, relevant for sleep onset. A decrease in GABA function, however, could lead to excitation of wake-promoting neurons instead [49]. Although not directly related to insomnia per se, a recent GWAS of sleep duration found that *ABCC9*, important for K_ATP_ channels, was relevant, again pointing to an important role for ion channel function. When this gene was utilized in a model system approach (using *Drosophila*), RNA interference of *ABCC9* resulted in flies that were unable to sleep for the first 3 h of their night, providing additional evidence for *ABCC9*’s role in sleep [80]. Additionally, *5-HTTLPR* is related to the stress response and hyperarousal, and given some associations with insomnia, could reflect an arousal mechanism [58]. Taken together, these results suggest a mechanism of changes in neuronal excitability, which if it occurs in wake-promoting neurons (or sleep-inhibiting neurons) could lead to hyperarousal and difficulty sleeping, thus promoting insomnia. This might suggest that genetic risk for insomnia is more related to “local” vs. “global” sleep processes.

These mechanisms could also underlie a vulnerability to greater sleep reactivity (the level of sleep disruption an individual experiences following a challenge; often a stressful event [28]), which could also be related to extended hyperarousal/excitability and in turn influence insomnia. The extant literature supports sleep reactivity as an insomnia risk factor (e.g., [81]), with a recent prospective study expanding findings to indicate that not only did higher sleep reactivity scores predict development of insomnia, the contribution of this effect was larger than that of family history [82]. Sleep reactivity has also been shown to be independently related to depression symptoms, although this relationship was mediated by insomnia [83], further emphasizing the importance of considering sleep reactivity as a potential insomnia mechanism. There is also evidence that sleep reactivity is moderately heritable [28,84], and that its genetic influences overlap significantly with those of insomnia (genetic correlation = 0.55 (females) and 0.65 (males); [28]). Thus, in addition to serving as a predictor of insomnia, sleep reactivity shares genetic vulnerability with insomnia, supporting the hypothesis that the two phenotypes are related on a genetic level. It may be that the genes currently identified for insomnia, since they are involved in excitatory pathways, are instead related to sleep reactivity, but more research would need to be done to examine this. There is some evidence that genetic mechanisms may be through hyperarousal as a recent family study has shown that parents with higher levels of stress-related insomnia have offspring more likely to exhibit higher cognitive-emotional hyperarousal [84]. Investigation of specific genes involved in sleep reactivity may also provide insights into insomnia and represent a potential area for early intervention by identifying individuals at risk for the disorder.

There is still much to understand about the contributions of specific genes and their mechanisms to insomnia. As the field of statistical genetics enters the post-GWAS era, the use of new techniques and advances that improve upon identification of relevant genes and expand the analysis of genome-wide data will be crucial in advancing the genetics of insomnia. For example, genome-wide complex trait analysis (GCTA) has provided a method for using genome-wide data to calculate the heritability of a trait in a large sample of unrelated individuals [85]. This method, which produces a SNP-based heritability estimate from the additive effect of all available SNPs, is not without limitations (e.g., power) and it should be noted that SNP-based heritability estimates for many common psychiatric phenotypes have been lower than twin estimates, but GCTA does offer a novel approach for analyzing genomic data. To our knowledge, there are currently no published GCTAs specifically of insomnia phenotypes. However, one recent study conducted GCTAs of depression symptom clusters, which included insomnia. The SNP-based heritability of insomnia symptoms was 30% in this sample, with estimates for other depression symptoms ranging from 5% (anxiety) to 30% (appetite) [86]. This does align with current insomnia heritability estimates, but should be interpreted with caution given that insomnia is presented in the context of depression.

There are also several methodologies that permit examination of molecular genetic overlap across phenotypes. This may be especially relevant for insomnia, as twin studies demonstrate significant (>50%) overlap in the additive genetic contributions to insomnia and common internalizing psychopathology (depression, anxiety) across the lifespan (e.g., [36,41,42]), indicating that understanding molecular overlap with common psychopathology may aid in the identification of insomnia-related genes. Further, recent studies show overlap with other psychiatric traits such as alcohol use disorder and psychotic symptoms (e.g., [42,38]). The polygenic risk score (PRS) approach uses the results from a GWAS of phenotype A (discovery sample) to create a risk score that is then applied to the genotypic data for individuals with phenotype B (target sample) and used to predict phenotype B. PRS approaches have been successful for phenotypes such as schizophrenia, identifying overlap with phenotypes such as bipolar disorder [87,88]. As GWAS of insomnia improve in phenotype and sample size, it will be important to conduct PRS analyses with insomnia and psychiatric disorders to expand upon twin studies.

Another new method for comparing across traits, which only requires data at the level of GWAS summary statistics (i.e., the output from a GWAS, which consists of the SNPs analyzed, their effects, and *p*-values for the specific phenotype analyzed; this is not individual-specific data), is linkage disequilibrium score regression (LDSC) [89]. LDSC uses summary statistics and information on the LD structure of the population used to generate a heritability estimate (similar to GCTA), and can produce a genetic correlation across multiple phenotypes. Bivariate GCTA can also be run to obtain a correlation, but has the disadvantage of requiring full genomic data for all individuals. LDSC may also prove useful for insomnia: A recent study used LD score regression and found significant genetic correlations between sleep duration and type 2 diabetes (rG = 0.26) and schizophrenia (rG = 0.19), suggesting that genetic variants that contribute to these disorders may also be relevant for sleep duration [90]. Thus, conducting genetic correlation analyses for insomnia and other complex traits (psychiatric and non-psychiatric in nature) has the potential to inform gene-finding efforts.

Beyond identification of genes and examination of overlap, there are methods to follow up on GWAS findings. Gene and pathway analyses can be useful to examine SNPs and genes of interest from GWAS and can determine if there is evidence that specific pathways or genes involved in specific functions are enriched within the sample (e.g., using Ingenuity^®^ Pathway Analysis software; [91]). The programs and methods available are constantly changing, as bioinformatics techniques advance rapidly. Epigenetics, which encompasses alterations in the genome that are not due to base pair changes (e.g., methylation, RNA interference [10]) may also be of particular relevance to insomnia, given that environmental factors are also important in the etiology of the disorder. A recent review suggests that since epigenetic mechanisms may be involved in sleep regulation as well as the stress response, it is plausible that epigenetics are a significant contributor to insomnia risk and symptoms [92].

Another genetic approach that can inform on insomnia mechanisms and may have more direct applications to a clinical population is gene expression (i.e., to what extent certain genes are turned on or off under specific conditions). Lately, we are gaining new insight into insomnia and treatment outcomes based on results from gene expression studies. Gill and colleagues [93] compared gene expression profiles of veterans with and without insomnia, finding differential expression of 43 genes in insomnia. One gene in particular, Urotensin 2, was significantly down regulated, and represents a potential insomnia target, given its role in orexin regulation and rapid eye movement. Another study by the same group investigated gene expression changes in military personnel with insomnia, focusing on differences between individuals who did or did not experience improvements in sleep following three months of insomnia treatment [94]. Positive changes in immune-related genes (i.e., lower expression of inflammatory cytokines, increases in regulatory genes), implication of the ubiquitin pathway (via pathway analysis) and improvement in depression symptoms were all seen in the improved sleep group, providing insight into the molecular changes that occur with successful insomnia interventions. Further, Irwin and colleagues [95] examined the effects of several insomnia interventions (CBT-I, tai chi) on levels of inflammatory markers in a randomized control trial design using older individuals. Both interventions resulted in positive changes. More studies of gene expression, particularly those that can extend findings to different populations with insomnia, will be useful in understanding molecular mechanisms of the disorder and how to treat it.

The goal of studies on genetic underpinnings of insomnia is to gain insight into the etiology of the disorder to inform development of interventions for both prevention and treatment of insomnia. For example, if we know that a certain gene influences an individual’s response to insomnia treatment (e.g., individuals with one genotype respond well to certain medication while those with other genotypes do not), we could screen individuals before treatment to maximize outcomes. The same strategy could also be used to determine whether an individual should receive cognitive behavioral therapy for insomnia or medication. Further, the identification of risk and protective genes could allow us to identify at risk individuals based on a combination of genotypes, allowing for early intervention and prevention. Eventually, it may even be possible to alter expression of risk genes (through drugs that target gene regulation) or use gene therapy to replace defective genes. There are many possibilities. Advances in psychiatric genetics have contributed to our knowledge of insomnia genetics, but there is still more work to do. Researchers should continue to focus on improving phenotypes and utilizing the most updated genetic methodology.

## Figures and Tables

**Table 1:** Overview of genetic methodology used within the insomnia literature.

Method	Brief Explanation	Output
Quantitative		
Family study	Compares prevalence of a phenotype in family members of affected vs. unaffected individuals.	Degree of familial resemblance.
Twin study	Utilizes the approximate genetic relationships between MZ and DZ twins to estimate the proportion of variance in a trait that is due to additive genetic (A), shared environmental (C), and unique environmental (E) influences.	Proportion of variance explained by genetic (i.e., heritability) and/or environmental effects.
Measured genes		
Candidate gene study	Gene to be examined are chosen *a priori* based on the literature. Association analyses are run to determine whether or not allele/genotype frequencies differ between cases and controls.	Strength of association (e.g., reported through beta or odds ratio) for variant(s) tested.
GWAS	Agnostic approach that simultaneously tests genetic associations across all available SNPs (often thousands to millions, depending on the method used).	Strength of association (e.g., reported through beta or odds ratio) for SNP(s) tested. Note that statistical corrections must be applied given the large number of tests.

Abbreviations: DZ = dizygotic; GWAS = genome-wide association study; MZ = monozygotic; SNP = single nucleotide polymorphism.

**Table 2:** Twin studies of insomnia phenotypes.

Author (Year)	Sample	Phenotype Ascertainment	Phenotype	Heritability
Adults				
Webb and Campbell, 1983 [18]	14 MZ, 14 DZ *	EEG	Sleep latency Wake time	*N*/A
Partinen et al. 1983 [19]	2238 MZ, 4545 DZ	Self-report (questionnaire)	Sleep length	*h*^2^ = 0.44
			Sleep quality	*h*^2^ = 0.44
Heath et al. 1990 [20]	1792 MZ, 2101 DZ	Self-report (questionnaire)	Sleep quality	*h*^2^ = 0.32
			Initial insomnia	*h*^2^ = 0.32
			Sleep latency	*h*^2^ = 0.44 (M), 0.32 (F)
			Anxious insomnia	h^2^ = 0.44
			Depressed insomnia	*h*^2^ = 0.44
Heath et al. 1998 [21]	1792 MZ, 2101 DZ	Self-report (questionnaire)	Composite score	12.1% of variance (F), 8.3% (M)
McCarren et al. 1994 [22]	1605 MZ, 1200 DZ male veterans	Self-report (questionnaire)	Trouble falling asleep	*h*^2^ = 0.28
			Trouble staying asleep	*h*^2^ = 0.42
			Waking up several times	*h*^2^ = 0.26
			Waking up tired	*h*^2^ = 0.21
			Composite score	*h*^2^ = 0.28
de Castro 2002 [23]	86 MZ, 129 DZ (“good sleepers“)	Self-report (sleep diary)	Sleep duration	*h*^2^ = 0.30
			Number of wakeups	*h*^2^ = 0.21
Watson et al. 2006 [24]	1042 MZ, 828 DZ *	Self-report (questionnaire)	Insomnia	*h*^2^ = 0.57
Boomsma et al. 2008 [25]	548 twins, 265 siblings	Self-report (questionnaire)	Insomnia factor	*h*^2^ = 0.20
Barclay et al. 2010 [26]	420 MZ, 773 DZ, 363 siblings *	Self-report (PSQI)	Subjective sleep quality	*h*^2^ = 0.41
			Sleep latency	*h*^2^ = 0.21
			Sleep duration	*h*^2^ = 0.00
			Habitual sleep efficiency	*h*^2^ = 0.30
			Sleep disturbances	*h*^2^ = 0.39
			Daytime dysfunction	*h*^2^ = 0.47
Hublin et al. 2011 [27]	1554 MZ and 2991 DZ	Self-report (questionnaire)	Insomnia-Symptom	*h*^2^ = 0.42 (M & F)
			Difficulty initiating sleep	*h*^2^ = 0.41
			Sleep latency	*h*^2^ = 0.41
			Nocturnal awakening	*h*^2^ = 0.45
			Early morning awakening	*h*^2^ = 0.34
			Non-restorative sleep (morning)	*h*^2^ = 0.37
			Non-restorative sleep (day)	*h*^2^ = 0.35
Drake et al. 2011 [28]	988 MZ, 1086 DZ	Self-report (DSM-IV-TR criteria)	Insomnia	*h*^2^ = 0.43 (M), 0.55 (F)
			Difficulty staying asleep	*h*^2^ = 0.25 (M), 0.35 (F)
			Non-refreshing sleep	*h*^2^ = 0.34 (M), 0.35 (F)
			Difficulty falling asleep	*h*^2^ = 0 (M & F)
Hur, Burri, and Spector 2012 [29]	893 MZ, 884 DZ, 204 individual twins	Self-report (questionnaire)	Insomnia symptom	*h*^2^ = 0.28
Genderson et al. 2013 [30]	339 MZ, 257 DZ, 26 individual twins	Self-report (PSQI)	PSQI global score	*h*^2^ = 0.34
			Subjective sleep quality	*h*^2^ = 0.31
			Sleep latency	*h*^2^ = 0.24
			Sleep duration	*h*^2^ = 0.29
			Habitual sleep efficiency	*h*^2^ = 0.23
			Sleep disturbances	*h*^2^ = 0.34
			Daytime dysfunction	*h*^2^ = 0.23
			“Poor sleep”	*h*^2^ = 0.31
Lind et al. 2015 [31]	Total sample *n* = 7500	Self-report (Symptom Checklist-90 sleep items)	Insomnia symptoms (Time 1)	*h*^2^ = 0.25
			Insomnia symptoms (Time 2)	*h*^2^ = 0.22
			Insomnia symptoms (longitudinal)	*h*^2^ = 0.59 (F), 0.38 (M)
Children				
Gregory et al. 2004 [32]	2162 MZ, 4229 DZ Age 3–7 years	Self-report (parents reporting on children)	Sleep problems scale	*h*^2^ = 0.18 (M), 0.20 (F)
Gregory, Rijsdijk and Eley 2006 [33]	100 MZ, 200 DZ Age 8	Sleep Self Report (completed by children), CSHQ (completed by parents)	Sleep onset delay	*h*^2^ = 0.17 (child report), 0.79 (parental report)
			Night wakings	*h*^2^ = 0.27 (child report), 0.32 (parental report)
Gregory et al. 2006 [34]	192 MZ, 384 DZ Age 8	CSHQ (completed by parents)	Sleep problems score	*h*^2^ = 0.61
Gregory 2008 [35]	100 MZ, 200 DZ Age 8	CSHQ (completed by parents)	Dyssomnia scale	*h*^2^ = 0.71
Gehrman et al. 2011 [36]	689 MZ, 666 DZ Age 8–16	Interview (sleep module of the CAPA)	Insomnia symptoms	*h*^2^ = 0.31
Moore et al. 2011 [37]	270 MZ, 246 DZ Avg age 12	Self-report (Child Behavioral Checklist, Youth Self-Report)	Sleep problems	*h*^2^ = 0.30
Taylor et al. 2015 [38]	1722 MZ, 1553 DZ Age 16	Self-report (Insomnia Severity Index and PSQI)	Sleep composite scores	*h*^2^ = 0.41
Barclay et al. 2015 [39]	739 MZ, 672 DZ Ages 8, 10, 14, 15	Clinical interview (CAPA)	“Clinically significant insomnia”	*h*^2^ = 0.14 (Wave 3)

Abbreviations: CAPA = Child and Adolescent Psychiatric Assessment; CSHQ = Children’s Sleep Habits Questionnaire; DSM-IV-TR = Diagnostic and Statistical Manual for Mental Disorders, Fourth Edition, Text Revision; DZ = dizygotic; EEG = electroencephalography; MZ = monozygotic; PSQI = Pittsburgh Sleep Quality Index. * Indicates a young adult sample.

**Table 3:** Genome-wide association studies (GWAS) of insomnia phenotypes.

Author (Year)	Sample	Phenotype Assessment	Insomnia Phenotype (s)	Main Findings
Ban et al. 2011 [68]	Korean epidemiologic study, total *n* = 8719 (1439 cases, 7280 controls)	Self-report (questionnaire)	Insomnia (self-report)	No genome-wide significant results. Top SNPs included rs11208305 (*PCLB1*; prior associations with schizophrenia) and rs718712 (*ROR1*; prior associations with bipolar).
Byrne et al. 2013 [69]	Australian twin registry, total *n* = 2323	Self-report (questionnaire)	Insomnia factor score, sleep latency, sleep time, sleep quality, sleep depth, sleep duration	No significant SNPs for insomnia factor score or other phenotypes. Of interest for sleep latency was rs7316184 (*CACNA1C*; prior associations with bipolar and schizophrenia), which did not replicate.
Parsons et al. 2013 [70] *	G1219 British sample, total *n* = 952	Self-report (PSQI)	Sleep quality, sleep duration, sleep latency (from the PSQI)	The *CACNA1C* gene was replicated, with rs16929277 associated with sleep quality in all models. This SNP was also associated with sleep latency and depression symptoms in recessive models. For the *ABCC9* gene, rs11046209 was only associated with sleep duration in a recessive model with rare genotypes, and rs11046205 was associated with depression symptoms.
Spada et al. 2016 [71]	LIFE Adult Study, Germany, total *n* = 956	Actigraphy	14 actigraphy parameters (e.g., sleep quality, sleep latency)	Significant SNPs: rs75842709 (*UFL1*; sleep efficiency on weekdays), chr9:865201D (*DMRT1*; sleep latency), and rs2919869 (*SMYD1*, sleep offset). Nominally significant SNPs: rs74448913 *CSNK2A1*; sleep latency) and rs12069385 (*ZMYM4*; sleep latency).
Amin et al. 2016 [72]	7 European cohorts, total *n* = 4242	Self-report (Munich Chronotype Questionnaire)	Sleep latency	Three correlated SNPs (rs9900428, rs9907432 and rs7211029) in *RBFOX3* were significant and could be replicated. Functional analyses indicated that this gene may be involved in GABA and monoamine release.

Abbreviations: PSQI = Pittsburgh Sleep Quality Index; SNP = single nucleotide polymorphism. * Indicates a replication.
